# Supplementary material for: Association of Seasonal Climate Variability and Age-Specific Mortality in Northern Sweden before the Onset of Industrialization
Source: Int J Environ Res Public Health. 2014 Jul 7;11(7):6940–54. doi: 10.3390/ijerph110706940 (PMC4113854; doi:10.3390/ijerph110706940)
Supplement: Supplementary File 1 — Supplementary Information (PDF, 126 KB) [file ijerph-11-06940-s001.pdf]

OPEN ACCESS

International Journal of
Environmental Research and Public Health
ISSN 1660-4601
www.mdpi.com/journal/ijerph

Supplementary Information

Association of Seasonal Climate Variability and Age-Specific Mortality in Northern Sweden before the Onset of Industrialization

Joacim Rocklöv [1], Sören Edvinsson [2], Per Arnqvist [3], Sara Sjöstedt de Luna [3] and Barbara Schumann [1,4,*]

[1] Centre for Global Health Research, Department of Public Health and Clinical Medicine, Umeå University, Umeå 90187, Sweden; E-Mail: joacim.rocklov@umu.se;

[2] Demographic Database, Umeå University, Umeå 90187, Sweden; E-Mail: soren.edvinsson@ddb.umu.se

[3] Department of Mathematics and Mathematical Statistics, Umeå University, Umeå 90187, Sweden; E-Mails: per.arnqvist@math.umu.se (P.A.); sara.de.luna@math.umu.se (S.S.L.)

[4] Ageing and Living Conditions Programme, Umeå University, Umeå 90187, Sweden

* Author to whom correspondence should be addressed; E-Mail: barbara.schumann@umu.se; Tel.: +46-90-786-62-42; Fax: +46-90-785-13-33.

This supplementary materials support the main text by providing sex and age-specific results. Table S1 shows total, sex- and age-stratified relative risks by annual climate variability. In table S2, total and sex-stratified relative risks by seasonal climate variability are shown.

Table S1. Annual climate variability and annual mortality: Total, sex- and age-stratified associations, Skellefteå 1749–1859.

				**Age Groups (Years), Men + Women**						
	**All**	**MEN**	**WOMEN**	**<1**	**1–2**	**3–9**	**10–14**	**15–24**	**25–49**	**≥50**
	**RR (CI)**	**RR (CI)**	**RR (CI)**	**RR (CI)**	**RR (CI)**	**RR (CI)**	**RR (CI)**	**RR (CI)**	**RR (CI)**	**RR (CI)**
**Annual temperature**	*0.94* *(0.88–1.01)*	*0.94* *(0.87–1.01)*	0.95 (0.88–1.02)	1.00 (0.93–1.07)	0.99 (0.88 1.12)	0.86 (0.69–1.08)	**0.76** **(0.61–0.94)**	0.89 (0.77–1.02)	*0.92* *(0.83–1.01)*	**0.91** **(0.85–0.97)**
**Annual precipitation**	1.00 (0.99–1.01)	1.01 (1.00–1.01)	1.00 (0.99–1.01)	1.00 (0.99–1.00)	1.00 (0.99–1.01)	1.00 (0.98–1.02)	1.00 (0.97–1.02)	1.01 (0.99–1.02)	*1.01* *(1.00–1.02)*	**1.01** **(1.00–1.02)**

Adjusted model: Annual temperature and precipitation adjusted for each other; RR, relative risk; CI, 95% confidence interval; RR indicate mortality risk by 1 °C increase in temperature resp. 1 cm increase in precipitation; Numbers in **bold**: significant (*p*-value < 0.05); numbers in *italics*: borderline significant (*p*-value < 0.10); Temperature: Mean temperature in °C; Precipitation: Cumulative precipitation in cm.

Table S2. Seasonal climate variability and annual mortality: Total and sex-stratified associations, Skellefteå 1749–1859.

	**Total Mortality**				**Mortality (Men)**		**Mortality (Women)**	
	Simple season-wise model *		Fully adjusted model **		Fully adjusted model **		Fully adjusted model **	
	RR (CI)	*p*-value	RR (CI)	*p*-value	RR (CI)	*p*-value	RR (CI)	*p*-value
Winter temperature	**0.97** **(0.95–0.99)**	**0.007**	*0.98* *(0.95–1.00)*	*0.057*	**0.97** **(0.95–1.00)**	**0.033**	0.98 (0.96–1.01)	0.137
Winter precipitation	1.00 (0.97–1.03)	0.783	0.99 (0.95–1.02)	0.343	0.99 (0.96–1.02)	0.567	0.98 (0.95–1.01)	0.217
Spring temperature	**0.95** **(0.91–0.98)**	**0.005**	0.96 (0.91–1.01)	0.103	0.97 (0.92–1.02)	0.248	*0.95* *(0.90–1.00)*	*0.050*
Spring precipitation	0.99 (0.97–1.01)	0.200	*0.98* *(0.97–1.00)*	*0.085*	0.99 (0.97–1.01)	0.142	*0.98* *(0.96–1.00)*	*0.064*

Table S2. *Cont.*

	**Total Mortality**				**Mortality (men)**		**Mortality (women)**	
	Simple season-wise model *		Fully adjusted model **		Fully adjusted model **		Fully adjusted model **	
	RR (CI)	*p*-value	RR (CI)	*p*-value	RR (CI)	*p*-value	RR (CI)	*p*-value
Summer temperature	1.01 (0.95–1.08)	0.692	1.04 (0.97–1.10)	0.300	1.02 (0.95–1.10)	0.541	1.05 (0.98–1.12)	0.173
Summer precipitation	*1.01 (1.00–1.03)*	*0.050*	1.01 (0.99–1.02)	0.294	1.01 (0.99–1.02)	0.431	1.01 (1.00–1.02)	0.226
Autumn temperature	0.99 (0.94–1.04)	0.674	1.00 (0.94–1.05)	0.898	1.00 (0.94–1.05)	0.861	1.00 (0.94–1.06)	0.941
Autumn precipitation	1.01 (1.00–1.02)	0.120	**1.02 (1.00–1.03)**	**0.036**	**1.02 (1.00–1.03)**	**0.033**	*1.02 (1.00–1.03)*	*0.063*

* Simple model: Seasonal temperature and precipitation adjusted for each other; each season separately; ** Fully adjusted model: Seasonal temperature and precipitation adjusted for each other; all seasons simultaneously included. RR, relative risk; CI, 95% confidence interval. RR indicate mortality risk by 1 °C increase in temperature resp. 1 cm increase in precipitation. Numbers in **bold**: significant (*p*-value <0.05); numbers in *italics*: borderline significant (*p*-value <0.10). Temperature: Mean temperature in °C. Precipitation: Cumulative precipitation in cm.

© 2014 by the authors; licensee MDPI, Basel, Switzerland. This article is an open access article distributed under the terms and conditions of the Creative Commons Attribution license (http://creativecommons.org/licenses/by/3.0/).
